# Gender Equality in Employment among Health Care Undergraduate Students: A Cross-Sectional Survey

**DOI:** 10.3390/healthcare10030543

**Published:** 2022-03-15

**Authors:** Chia-Jung Hsieh, Kai Pan, Hui-Chen Lee, Ya-Ling Shih

**Affiliations:** 1 School of Nursing, College of Nursing, National Taipei University of Nursing and Health Sciences, Taipei 112303, Taiwan; huichenl@ntunhs.edu.tw (H.-C.L.); tiffanysyl0902@gmail.com (Y.-L.S.); 2 Department of Infant and Child Care, College of Human Development and Health, National Taipei University of Nursing and Health Sciences, Taipei 112303, Taiwan; kaipan@ntunhs.edu.tw

**Keywords:** gender equality, health care field, health care undergraduate student

## Abstract

Objectives: The purposes of this study were to predict the important variables associated with health care undergraduate students’ opinion of gender equality (GE) in employment. Design: This study adopted a cross-sectional design with multistage sampling and adhered to STROBE guidelines. Setting: From one of the universities of health care in northern Taiwan. Participants: We recruited 2855 health care students and the questionnaire response rate was 78.3%. Results: Female students in this study have a better concept of GE in employment. There was a statistically significant negative correlation between the subjects’ gendered concept of family and GE in employment. From the results of multiple linear regressions, the important and significant variables were female, sophomore students, students who were in the division of continuing college self-attitude toward gender roles, attitude toward parental expectations of gender roles, those could explain 45.3% of the variation in GE in employment. Conclusion: Students exposed to the active cultivation of GE in health education largely benefit future professional development. Establishing a GE concept on campus will help students develop their profession in the future. Encourage the school to offer at least one “gender-related” elective subject per semester, especially in the on-the-job training programs to satisfy the needs of GE education.

## 1. Introduction

Gender equality (GE) is essential for a civically literate global citizen and a growing trend that prohibits discrimination in employment worldwide. Since the beginning of the feminist movement in the 1960s, social development has progressed towards gender equality and gender equity. Many people use these two names interchangeably; however, there is a distinction between the two terms [1]. Gender equality is a straightforward concept but more problematic when measured in terms of outcomes, and gender equity is about perceptions of fairness and opportunity rather than strict equality of outcome, it allows for different outcomes for men and women so long as both parties regard them as fair or at least not grossly unfair [2]. However, the international community adopted a United Nations resolution on 2030 and Sustainable Development, including a set of Sustainable Development Goals (SDGs); it contains several commitments to gender equality [3]. Gender equality in health is the belief that women, men, and LGBT (lesbian, gay, bisexual, and transgender) people should have equal opportunities for realizing their full rights and potential to be healthy and contribute to health [4].

Previous studies on college students have revealed a more advanced concept of gender equality [5]. Some cross-cultural studies pointed out that males expressed stronger sex-role stereotypes than females did [6,7], and with different values in the self-concept of college students [6]. Girls and boys perceived the gender stereotypes about computing held by their parents, and such attitudes inversely affected the female students’ attitudes [8].

The fact that gender stereotypes may impact patient rights or nurse–patient relationships or contribute to gender disparities in healthcare [4] shows that gender equality in the workplace is crucial for students who aim to devote themselves to the health care field [9]. Hence a gender-friendly environment is necessary for the health care industry for the student to cultivate sensitivity to gender differences and be aware of gender equality issues [10]; Medical staff can also play an important role in reducing gender discrimination and role pressures, as well as promoting better nurse-patient communication and holistic nursing [9,11].

However, in academic medicine, gender inequality and inequity have persisted [12]. Lack of the concept of GE could result in stereotypes, over-simplification, or generalization of gender roles, and ignoring individual differences. The most common phenomenon resulted in a dichotomy between males and females, in terms of personality, attitudes, lifestyles, career preferences, and polarization of employment [13]. Hence, a recent study also pointed out that incorporating gender issues into the classroom could increase students’ awareness of gender equality in universities’ general curriculum [14]. A recent World Bank report also pointed out that reducing gender inequality makes economic sense apart from being the right thing to do because gender inequality affects an individual’s life and leads to the loss of human capital wealth [15].

Many countries have been committed to the practice of GE in a wide array of actions, which already include a significant focus on gender equality in governing regulations and management structures [16]. Based on the policy and strategy promoted by the international community, Taiwan has also passed the Employment Gender Equality Act and the Gender Equality Education Act, respectively [17,18]. The former law was implemented in 2002 and aimed to establish an equal working environment [17]. The latter act was implemented in 2004 and is committed to the implementation of educational institutions at all levels, aiming to promote true gender equality, eliminate gender discrimination, maintain human dignity, and improve representative educational resources and environments [18].

Starting from the awareness of gender identity, students can learn how to accept and value different gender categories, eliminate gender stereotypes, and refrain from any gender prejudice or discrimination [10,18]. Review of the literature revealed that the topics of current gender equity education-related courses in the colleges and universities are very diverse. Still, only 4.5–5.1% of total college students took the courses, and most of them focused on “gender education for the child”, “gender relations”, and “sex education” [19]. Few of the studies were targeted on equal opportunities in employment, not to mention the enrollment of students in the health care departments.

Previous studies have reported implicit gender stereotyping of school as a predictor of academic achievement, i.e., the more feminine characteristics or female orientation in the courses, the more male students ascribed negative masculine traits to themselves [20] For example, in nursing education, males tend to have a sense of isolation when they step into the nursing field; moreover, female educators are prone to sexually stereotype male students, scrutinize their moves, and treat them negatively or contradictorily [21,22]. Therefore, the educators should be able to guide the students to respect the choice of others via self-examination and self-reminding innate differences among various gender identities. Understanding and recognizing gender diversity will be beneficial to the development of healthcare professionals [10,23]. Qualitative research of male nurses in clinical work has reported that they are susceptible to outsiders’ curiosity, eye-catching and given high expectations toward them in the work [24]. In addition, there is a kind of prejudice, which is easy to be ignored and invisible. It is called “benevolent sexism”. This discrimination refers to viewing different gender roles in a stereotyped and fixed role; on the surface, it seems to love the individual, but it is rooted in traditional male-dominated beliefs, and the results can also be harmful to the individual [25], i.e., evaluations of men or women that are seemingly positive, for example, reverence of men in the nursing workplace due to fewer household chores and potential as a manager, and qualities stereotypically assigned to females such as warm, soothing, sensitive, etc., all the above could weaken the promotion of gender equality [26]. The belief that men have a duty to protect women, another example of benevolent sexism, could also dampen the enthusiasm [27]. Furthermore, such discrimination is easily neglected [21]; those who had stronger gender stereotypes showed a weaker perception of nursing professionalism, which can hamper their growth as professionals [11]. Taken together, to reach the goal of gender equality in employment, it is necessary to highlight the concept of gender equality in right-to-work and to prevent gender discrimination in school education.

Consequently, the following hypotheses for this study were developed as a result of the literature review [6,7,8,14]. (1) The concept of gender equality in employment was affected by the basic attribute of research subjects (parental expectations and self-attitudes toward gender roles); (2) There was a significant correlation between the courses the subject has taken and the concept of gender equality; (3) Significant predictors of gender equality in employment were variables of gender, self-attitudes toward gender roles, parental expectations of gender roles, and gender equality course enrollment.

### Aims and Objectives

The aims of this study were as follows. (1). To explore the concept of gender equality (GE) in employment for health care undergraduate students; (2). To investigate the effects of the basic demographic data of the participants on the GE in employment; (3). To predict the important variables associated with undergraduate students’ opinion of *GE* in employment. The result of this study portrays the basis for evaluation of the gender equality movement in nursing education and stands for a future reference for curriculum design.

## 2. Methods

### 2.1. Design

This study adopted a cross-sectional design with multistage sampling and adhered to the Strengthening the Reporting of Observational Studies in Epidemiology (STROBE) guideline (see Supplementary Material Table S1).

### 2.2. Sites and Participants

The students in a university of nursing and health science in northern Taiwan were enrolled. Originally, a multistage sampling was designed, i.e., the students in this university were assigned as the population; in the first stage, the population was divided into three colleges. For the next stage, different grades were randomly sampled from each department, and G power software developed by Faul, Erdfelder, Lang, and Buchner [28] was applied to estimate the sample size via regression analysis (alpha = 0.05, power = 0.95, effect size *f*^2^ = 0.02, small effect size), based on the fact of 13 independent variables, the number of samples must be at least 1339. As an expected rejection rate at 20%, 1610 participants should be enrolled. However, due to the fact that this study was a Ministry of Education (MOE) granted carried out by the gender equality education committee, the population census was applied instead, and a total of 3646 questionnaires were issued. The modification was reviewed and approved by the JIRB.

The inclusion criteria were as follows: (1) age at least 18 years, signed informed consent (agreement of parents to provide informed consent for those younger than 20 years old); (2) school ID provided, currently registered as evidenced by the registration stamp of this semester. Exclusion criteria are as follows: (1) not registered in the current semester (including suspension or transfer); (2) refusing to participate in this study.

**Figure 1:**
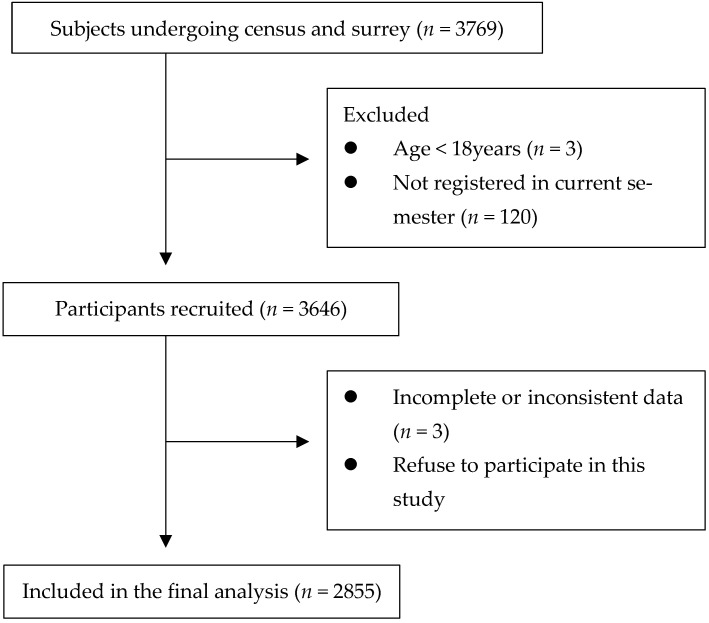
Flow chart of participant recruitment.

After sample collection and removal of blank and invalid questionnaires (the same answer for positively and negatively worded tests), 2855 valid samples were recovered for data analysis. The valid response rate was 78.3%. (Figure 1)

### 2.3. Ethical Considerations

This study was reviewed and approved by the Joint Institutional Review Board (JIRB No. 16-S-010-1). Each respondent provided written informed consent before inclusion in the study.

### 2.4. Measurements and Demographic

The basic attributes of the student include gender, age, college, school system, department, grade, experiences in gender course enrollment and a number of courses enrolled.

### 2.5. Attitude toward Parental Expectations of Gender Roles

The five-item questionnaire was developed from the literature review [29,30] with the main assessment of family (father and mother) images of gender roles the attitude of nuclear family members toward the cultivation of sons. The higher the score, the more severe the gender stereotype in the family. The content validity of this questionnaire was carried out by five experts (5-point scale), and with an expert CVI of 0.91. The reliability test revealed Cronbach’s alpha value = 0.87.

### 2.6. Self-Attitude toward Gender Roles

The self-evaluate, three-item questionnaire for the students was developed from the literature review [31,32] with the main assessment of the subjects’ acceptance of self-gender with a six-point scale. The higher the score, the more positive the self-attitude toward gender roles. The content validity of this questionnaire was carried out by five experts (five-point scale), and with an expert CVI of 0.94. The reliability test revealed Cronbach’s alpha value = 0.78.

### 2.7. Survey of Gender Equality in Employment

This scale, as developed by Chen [33] includes 35 items and is categorized into six attributes, i.e., “competent as manager”, “gender stereotype in employment”, “dedication and commitment to work”, “job performance and capability”, “role conflict and double-income family”, “promotion and training opportunities, salary and employment”. In the positively worded questions, “strongly agree”, “generally agree”, “somewhat agree”, “somewhat disagree”, “generally disagree”, “strongly disagree” scored 6, 5, 4, 3, 2, and 1, respectively, and vice versa. A higher score indicates better concept of gender equality in employment. A reliability of the scale is revealed as an internal consistency at 0.89. The construction validity in factor analysis is also in the acceptable range [33]. Cronbach’s α 0.95 indicated good reliability of this scale.

### 2.8. Research Procedures and Data Collection

There are four stages in the procedure, i.e., development of expert validity, pre-test, the real test, and data analysis and report preparation. Data was collected from September 2016 to January 2017. 20 to 30 min is required to complete each questionnaire. After the classes of the participants were identified, the research team explained the procedure of this study during their class meeting. After the agreement, the participants could decide either to complete the questionnaires on the spot or at home. The students could sign their informed consent by themselves (for those under 20 years old, informed consent from their parents is also required). The participants were also informed of their rights to voluntarily withdraw the consent to participate in this study and with their student rights not affected. The research team employed a number to keep each participant’s identity confidential. After the completion of all questionnaires, the cadre in the class collects all the questionnaires in a large envelope and submit them to the gender equality office. For those who complete the survey at home, they could hand it in the mailbox of the gender equality office.

### 2.9. Statistical Analysis

After data collection and filing, the IBM SPSS software package (Version 21.0, Armonk, NY, USA) was applied for descriptive and inferential statistics, including *t*-test, variance analysis (post-test when significance was found), Pearson product-moment correlation, multiple regression analysis, etc. [34]. The significance level was set at α value of 0.05.

### 2.10. Patient and Public Involvement Statement

As this study focused on health care students, neither patients nor the public had roles in the design, collection, analysis, and interpretation of data or in writing the manuscript.

## 3. Results

### 3.1. Basic Attributes of Research Subjects

A total of 2855 subjects participated in this study, of which 15.7% are males, with an average age of 21.72 ± 2.96 years old. Most of the participants were in the college of nursing, and the majority (67.85%) were four-year college students. 89% did not take any courses related to gender in the university; the average number of courses taken was 0.09 ± 0.31 courses (Table 1).

### 3.2. The Scores in the Attitudes toward Parental Expectations for Gender Roles as Well as Gender Quality in Employment

The average score in the attitudes toward parental expectations for gender roles was 2.25 ± 0.98; among them, the feeling of “male family members do less housework” scored the highest. The average score in the attitude toward gender equality in employment was 4.84 ± 0.65, with the highest score found in “promotion and training opportunities, salary and employment” (Table 2).

### 3.3. Contribution of the Research Subject’s Basic Attributes to Their Attitudes toward Parental Expectations for Gender Roles as Well as GE in Employment

Results indicated significant difference in the attitudes toward parental expectations for gender roles was found in the gender, i.e., score in female higher than that in male (*t* = −2.7, *p* < 0.01), school system (*F* = 33.29, *p* < 0.001) and grade (*F* = 23.5, *p* < 0.001). In other words, female students in the higher grade of on-the-job training system expressed more obvious stereotype in terms of parental expectations for gender roles (table omitted).

In addition, our results unraveled higher score of gender equality employment in females than that in males (*t* = −11.18, *p* < 0.001); significant difference in gender equality employment was also found in the college categories (*F* = 9.62, *p* < 0.001), school systems (*F* = 12.45, *p* < 0.001) and grade (*F* = 9.24, *p* < 0.001). Post-hoc analysis unveiled poor score of gender equality in employment in male students in the on-the-job training system, and better performance was found in first-grade students (Table 3).

### 3.4. Pearson Product-Moment Correlation Analysis of the Attitudes towards Gender Equality in Employment

Here we showed significant negative correlation between gender equality in employment in both age (*r* = −0.10, *p* < 0.001) and attitude toward parental expectations of gender roles (*r* = −0.20, *p* < 0.001). Significant positive correlation was found between gender equality in employment and both the number of gender-related courses enrolled (*r* = 0.04, *p* < 0.05) and self-attitude toward gender roles (*r* = 0.23, *p* < 0.001) (Table 4).

### 3.5. Best Predictors of Perceptions of Gender Equality in Employment

Results from multiple regression analysis showed significant associations between the perceptions of gender equality in employment and female (*β* = 0.234; *p* < 0.001), sophomore students (*β* = −0.058; *p* < 0.001), continuing program (*β* = −0.149; *p* < 0.001), self-attitude toward gender roles (*β* = 0.112; *p* < 0.001), attitude toward parental expectations of gender roles (*β* = −0.015; *p* < 0.001), taken together explained 45.3% of the variance (Table 5).

## 4. Discussion

### 4.1. Research Subjects’ Perceptions of Gender Equality in Employment

We showed in this study that among the perceptions of “attitudes toward parental expectations on gender roles”, the highest score was found in “males did less housework in my family” and “my college planning was based on my family’s recommendation for my future career development”. This finding highlights hidden gender stereotype in real-life experiences, i.e., biased housework sharing in the private domain, albeit the continuous promotion of gender equality education. The result is consistent with the description of dividing household chores between male and female members in the Gender Equality Policy Guidelines [17], i.e., despite domestic universalization of education and increase in labor force participation have weakened traditional gendered division of labor, women continued to carry heavy household and family care duties, either actively or passively.

According to the National Development Council [35], despite the fact that the male participation rate in housework has increased significantly, the implementation of gender equality in household chores in the private domain was still insufficient. It has been argued that the under-representation of women is the result of unequal opportunities, gender stereotypes and prejudice [36]. In addition, previous studies have shown that the upbringing of an individual greatly shaped their values on gender as well as the segregation of gender identity [37], which again reflected the long-term effect of parental or nuclear member’s perception of gender roles in the cultivation of gender identity of an individual. We showed in this study that in terms of the attitude toward gender equality, the highest scores were found in the roles in the public domain, such as “opportunities of promotion and training, salary and employment”, which indicated positive thinking in most of the participants regarding the female employment, i.e., fair opportunities for both genders in the workplace.

### 4.2. The Impact of the Basic Attributes on GE in Employment

Hypothesis (1) was proven to be accurate, i.e., the concept of gender equality in employment is affected by the basic attribute of research subjects (parental expectations for gender roles, gender, age, school system, and self-attitudes toward gender identity), and described as follows.

The role of the family in gender identity development has been demonstrated that an individual’s subjective consciousness was cultivated via observation and simulation in daily life since childhood. On the other hand, previous studies also showed gradual attenuation of parental influence as the increase of socialization experiences, the role model for their child was limited to early adolescence [36]. Our results demonstrated higher scores of perception of parental expectations on gender roles in female students as compared to male students, indicating stronger gender stereotypes in family life in female students. Nevertheless, our results also showed more advanced perception of gender equality in female than male students, which is consistent with the results of other domestic studies. Weng et al. [30] reported when compared to boys; girls had a better sense of gender equality in “gender stereotype”, “gender prejudice”, “gender roles,” and “gender identity”.

According to stereotypical beliefs about the sexes, women are more communal (selfless and concerned with others) and less agentic (self-assertive and motivated to master) than men [38]. Tzeng [37] found the promotion of feminism and challenges of traditional patriarchal values may result in more traditional male students in gender equality and flexibility than females, and strengthened women’s attention toward gender equality issues, and hence better self-identification, gender and awareness, opposition to rigid gender roles, and breaking the shackles of gender bias. Furthermore, studies also revealed a significantly positive correlation between the value the individual placed on self-gender identity and the perception of gender equality, i.e., the more positive attitude toward self-gender identity, the better the concept of gender equality.

### 4.3. The Relationship between Course Enrollment and GE in Employment

Nearly 90% of the participants in this study did not enroll in gender-related courses during their college years. Further analysis revealed the percentage of male and female students in gender-related course enrollment was 4.90% and 12.11%, respectively, indicating more active learning of gender equality in employment in females than that in males [30,37], but also underlined the importance of continuous promotion of gender equality education and a more in-depth evaluation of the applicability of current education practice to different genders for how to improve male enrollment in gender-related courses, such as to motivate the learning, to create a friendly learning environment, etc.

Hypothesis (2) was proven statistically. A significantly positive correlation was found between the courses taken and the concept of gender equality, i.e., those who have enrolled in gender-related courses tend to favor gender equality, and the more courses they took, the better concept of gender equality in employment. Liu [18] reported active cultivation of gender equality awareness for the students is indispensable in general gender education. Through values clarification, students were able to view their gender roles and enhance self-awareness [23,39]. Our study also revealed an intriguing finding in a positive correlation between the course enrollment and attitude toward parental expectations of gender roles; a possible explanation could be that students tend to choose gender equality courses of their own free will, especially when they sensed more gender stereotypes in the family, which is consistent with the finding of Wu and Wang [10] the importance of creating a gender-friendly nursing education environment, and gender equality education should be integrated into the nursing education and incorporated into clinical practice.

### 4.4. Best Predictors of GE in Employment

Hypothesis (3) was also statistically proven. We unraveled in this study the higher the score in the perception of stereotypical parental expectations of gender roles, the worse the attitude toward gender equality, and the effect was significant. Our finding is consistent with the result of Weng et al. [30] that for college students, parenting attitudes was positively correlated with as well as best predicted the awareness of gender equality, probably caused by the consolidation of benevolent sexism in the family and awaits future change of the concept in the private domain [26]. Nevertheless, perception of gender equality was also influenced by personal attitudes as the better concept of gender equality was found in women, and the younger the better the concept, which could explain the achievement of gender equality education in students in the lower grades in recent years. For the students under the on-the-job training programs, gender equality education may be limited in the previous college system. More enrollments may improve it in gender education courses in the university level [40]. Gender equality education is necessary for healthcare professionals in enhancing their gender sensitivity [41]; therefore, it is indispensable to incorporate gender analysis and gender equality courses into the healthcare education program.

### 4.5. Limitation

The limitations of this study are as follows. The participants came from the same university, and hence the conclusion is not representative of all college students. Most of the participants are females, which potentially limits the generalizability of findings. Due to the cross-sectional design, the conclusion is limited to the attitude toward gender equality in employment in the particular time window and is challenging to investigate causality. On the other hand, the high nonresponse rate of questionnaires, i.e., 21.7%, impacted the inference of this study. Considering this, we acknowledge a possible positive response bias because only the most motivated students (who are also presumably more sensitized to gender equality issues) might have responded to our questionnaire.

In addition, only the gender-related courses enrolled in the university years were investigated here; future studies encompassing experiences in all gender-related courses taken are required for more comprehensive conclusions.

## 5. Conclusions

To our knowledge, this is the first study of gender equality in employment focusing on students in the health care field. The results indicated gender, age, attitudes toward parental expectations, and self-awareness of gender roles are key elements affecting the perception of gender equality in employment. A higher score of perception of gender equality in employment was found in female college students, and albeit the experience of gender stereotype from the family, the participants were positive about female participation in the workplace, and both genders derive equal opportunities in employment. A significant correlation was found between the number of gender-related courses enrolled and the perception of gender equality. Taken together, the active cultivation of gender equality in health education largely benefits future professional development. Here are our recommendations regarding curriculum and activities, the professional preparation of teachers, and future research directions.

Curriculum and activities: (1) Encourage each school to offer at least one “gender-related” elective subject per semester, especially in the on-the-job training programs to satisfy the needs of gender equality education. It is also recommended to check and connect transversality in all subjects. (2) Re-inspection of current professional courses, not only the promotion of gender equality but also the re-assessment of hidden gender prejudice or stereotypes in teaching cases. (3) To understand the students’ prior experiences in gender equality education, conduct pre-assessments before the initiation of each gender-related course for the design of teaching activities. The pre-assessments also enable a preliminary evaluation of the students’ overall perception of gender equality. They are helpful in evaluating the effects of previous gender prejudice or stereotype on their physiological and psychological development. (4) Regular seminars or workshops on gender equality to facilitate the interaction between genders. Male-specific activities are also recommended for more specific teaching and discussion to further understand their opinions on gender-related issues.

Professional preparation of teachers: (1) Encourage peer support. Teachers with the concept of gender mainstreaming can share their teaching experiences in the communities of the faculty; empower educators to get the knowledge and skills they need through the training of seed teachers in each institution and department. (2) Encourage participation in conferences on gender equality to enhance the knowledge and more diversified learning from academic events.

## Figures and Tables

**Table 1:** Sample Profile (*n =* 2855).

Variable	*n*	Percent
Gender		
Male	449	15.70
Female	2403	84.20
Missing	3	0.10
Age (*mean ± SD)*	(21.72 *±* 2.96)	
College		
College of nursing	1427	50.00
College of health technology	714	25.00
College of human development and health	714	25.00
School system		
Four-year technology college	1937	67.85
Two-year technology college	652	22.83
Continuing college	266	9.32
Grade		
Freshman	651	22.80
Sophomore	485	17.00
Junior	927	32.50
Senior	792	27.70
Lesson experience of gender-related		
Yes	313	11.00
No	2542	89.00
Number of gender-related courses enrolled (*mean ± SD)*	(0.09 *±* 0.31)	

**Table 2:** The attitudes toward parental expectations for gender roles as well as gender quality in employment.

Variable	Mean	SD	Sequence
The attitudes toward parental expectations for gender roles	2.25	0.98	
I think my father had patriarchal attitudes.	1.99	1.26	3
I think my mother had patriarchal attitudes.	1.98	1.27	4
At home, male family members do less housework.	2.93	1.67	1
At home, the boys would think that priority should be cultivated.	1.82	1.11	5
I chose the subject I studied because my family believed that this career development was suitable for my gender development.	2.56	1.51	2
Attitude toward gender equality in employment	4.84	0.65	
Competent as manger	5.00	0.82	3
Gender stereotype in employment	5.04	0.82	2
Dedication and commitment to work	4.38	0.73	6
Job performance and capability	4.67	0.66	5
Role conflict and double-income family	4.92	0.78	4
Promotion and training opportunities, salary and employment	5.17	0.89	1
Self-attitude toward gender roles	4.91	0.89	

**Table 3:** The research subject’s basic attributes to their attitudes toward GE in employment (*n* = 2855).

Variable	Mean	SD	*F/t*	*p*
Gender			−11.18	<0.001
Male	4.49	0.73		
Female	4.90	0.61		
College			9.62	<0.001
College of Nursing	4.89	0.62		
College of Health Technology	4.76	0.69		
College of Human Development and Health	4.81	0.65		
School system			12.45	<0.001
Four-year technology college	4.83	0.65		
Two-year technology college	4.91	0.59		
Continuing college	4.68	0.63		
Grade		9.24	9.24	<0.001
Freshman	4.94	0.61		
Sophomore	4.74	0.69		
Junior	4.84	0.65		
Senior	4.81	0.64		
Lesson experience of gender-related			−1.35	0.179
Yes	4.83	0.65		
No	4.88	0.61		

**Table 4:** Pearson product-moment correlation analysis of the attitudes towards GE in employment (*n* = 2855).

Variable	1	2	3	4	5
1 Age	1				
2 Number of gender-related courses enrolled	0.11 ***	1			
3 Self-attitude toward gender roles	−0.02	0.05 **	1		
4 Attitude toward parental expectations of gender roles	0.19 ***	0.08 ***	−0.15 ***	1	
5. Attitudes towards GE in employment	−0.10 ***	0.04 *	0.23 ***	−0.20 ***	1

* *p* < 0.05. ** *p* < 0.01. *** *p* < 0.001.

**Table 5:** The multiple regression analysis of the attitudes towards GE in employment *(n =* 2855).

Variable	Unstandardized Coefficients		Standardized Coefficients		
	B	Standard Error	Beta	*t*	*p*
(Constant)	2.400	0.115		20.894	<0.001
Gender (Ref: Male)	0.234	0.026	0.132	8.980	<0.001
Grade (Ref: Freshman)					
Sophomore	−0.058	0.015	−0.068	−3.997	<0.001
Junior	−0.017	0.009	−0.037	−1.807	0.071
Senior	−0.008	0.008	−0.022	−0.996	0.319
College (Ref: College of Nursing)					
College of Health Technology	−0.101	0.024	−0.068	−4.250	<0.001
College of Human Development and Health	−0.102	0.024	−0.068	−4.298	<0.001
School system (Ref: Four-year)					
Two-year technology college	−0.023	0.027	−0.015	−0.858	0.391
Continuing college	−0.149	0.043	−0.067	−3.425	<0.001
Age	−0.006	0.004	−0.026	−1.308	0.191
Number of gender-related courses enrolled	0.007	0.031	0.003	0.215	0.830
Self-attitude toward gender roles	0.112	0.003	0.593	41.216	<0.001
Attitude toward parental expectations of gender roles	−0.015	0.002	−0.115	−7.964	<0.001

Note: Ref: Reference; Residuals: Maximum: 0.932; Minimum: 0.399; Residuals variance analysis was consistent with the assumption of homogeneity.

## Data Availability

The dataset generated during the current study is available from the corresponding author on reasonable request.

## Supplementary Materials

The following supporting information can be downloaded at: https://www.mdpi.com/article/10.3390/healthcare10030543/s1, Table S1: STROBE Statement—Checklist of items that should be included in reports of cross-sectional studies.
